# Inhibitory effect of standardized *Kaempferia parviflora* extract on sarcopenia by improving protein metabolism pathways in aged C57BL/6J mice

**DOI:** 10.1016/j.jtcme.2025.03.007

**Published:** 2025-03-14

**Authors:** Changhee Kim, Minseong Kang, Yeeun Kim, Jae-Kwan Hwang

**Affiliations:** a Department of Biotechnology, Yonsei University, Seoul, Republic of Korea; b Graduate Program in Bioindustrial Engineering, Yonsei University, Seoul, Republic of Korea

**Keywords:** *Kaempferia parviflora*, Sarcopenia, Protein turnover, Mitochondrial homeostasis

## Abstract

**Background:**

Sarcopenia is a muscle disease typically characterized by an age-related loss of muscle mass and function, leading to the deterioration of health in older adults. The current study aimed to investigate the therapeutic potential of standardized *Kaempferia parviflora* extract (KPE) on sarcopenia and elucidate its underlying molecular mechanisms.

**Methods:**

To assess the inhibitory effects of KPE on sarcopenia, 18-month-old C57BL/6J mice were employed as an aged model. The mice were orally administered 100 and 200 mg/kg KPE daily for 8 weeks.

**Results:**

KPE improved age-reduced grip strength and exercise endurance. It not only increased hindlimb muscle weight, volume, and myofiber cross-sectional area but also decreased abdominal fat volume and adipose tissue weight. KPE downregulated the mRNA expression of tumor necrosis factor alpha and interleukin-6 by inactivating nuclear factor kappa B protein expression and increasing the mRNA expression of anti-oxidant enzymes. Moreover, it significantly reduced the mRNA expression of muscle ring finger-1 and muscle atrophy F-box for proteolysis and stimulated the mammalian target of rapamycin pathway for protein translation by stimulating the phosphatidylinositol 3-kinase/Akt pathway. KPE also increased the relative mitochondrial DNA content by upregulating peroxisome proliferator-activated receptor gamma coactivator 1 alpha, nuclear respiratory factor 1, and mitochondrial transcription factor A mRNA expression.

**Conclusion:**

This study's findings demonstrate that KPE improves age-induced sarcopenia by regulating protein turnover and mitochondrial homeostasis. Therefore, KPE is potentially useful as a functional ingredient that inhibits or decelerates sarcopenia.

## Graphical abstract

**Figure:**
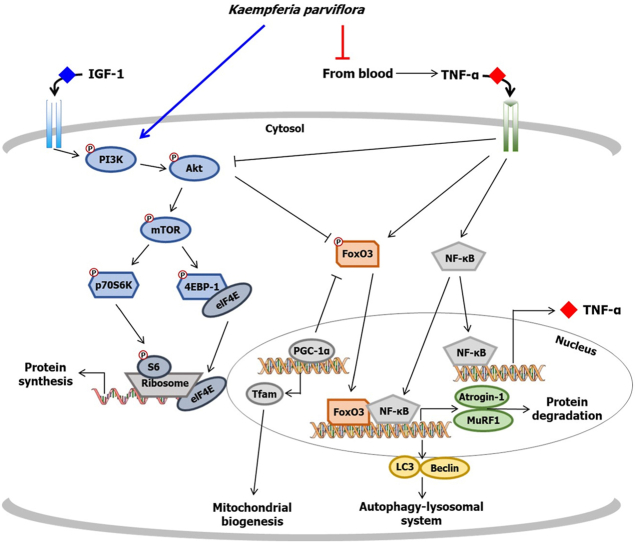

## 1 Introduction

Sarcopenia, an aged-related disease characterized by the progressive loss of muscle mass, is accompanied by declines in muscle strength and function.^1^ Along with a reduction in muscle mass and function, sarcopenia elicits other adverse outcomes, including physical disability and metabolic abnormalities, such as insulin resistance, leading to a poor quality of daily life and, eventually, death in older adults.^1^^,^^2^ Muscle mass decline commences at 20 years of age; 50% of adults aged >80 years are afflicted with sarcopenia.^3^^,^^4^ Unfortunately, owing to a greater global older population and increasing life expectancy, the prevalence and treatment cost of sarcopenia are rising and will become more socially burdensome.^3^

Several methods for treating muscle atrophy, including exercise training; electronic shock; and gene-, cell-, and chemical-based therapies, are being extensively explored.^2^^,^^3^^,^^5^ Regular exercise training, especially resistance exercise, is a well-known intervention for muscle atrophy. Unlike other therapies that induce adverse side effects or lack efficacy, resistance training is supported by clinical data and approved for therapeutic use.^1^^,^^3^ However, this intervention is yet to be widely adopted as a treatment for sarcopenia. Owing to weakness, frailty, illness, a sedentary lifestyle, and bed rest, older people and patients with sarcopenia are considerably limited in their ability to exercise regularly.^1^^,^^3^^,^^6^ Therefore, alternative and feasible options for these subpopulations should be developed.

Since sarcopenia is a multifactorial and systematic muscle disease, identifying its underlying mechanisms will provide insight for developing new molecular treatments.^1^^,^^7^ On this premise, numerous studies have focused on developing novel therapeutic targets for sarcopenia, emphasizing protein synthesis, protein degradation, mitochondrial homeostasis, the inflammatory response, and oxidative stress.^8^^,^^9^ Several studies have recently revealed the target molecular mechanisms of potentially therapeutic herbs and phytochemicals that may be used to combat muscle wasting. For example, apigenin increased the running distance and cross-sectional area of muscle fibers in an obesity model by improving mitochondrial function and decreasing the levels of tumor necrosis factor alpha (TNF-α) and interleukin-6 (IL-6), which are major proinflammatory cytokines that stimulate muscle wasting.^10^ Oligonol, a flavanol-rich lychee extract, has been shown to not only increase protein turnover by stimulating the Akt/mammalian target of rapamycin (mTOR) pathway but also stimulate mitochondrial biogenesis through peroxisome proliferator-activated receptor gamma coactivator 1 alpha (PGC-1α) in aged senescence-accelerated mouse prone 8 (SAMP8) mice, thereby increasing muscle mass and grip strength.^11^ Therefore, the consumption of easily accessible plant extracts and phytochemicals with minimal side effects is a potential intervention for patients with sarcopenia and older adults.

*Kaempferia parviflora* Wall. Ex. Baker, commonly known as black ginger, is a member of the Zingiberaceae family. Traditionally, this plant has been used as a food and an herbal medicine to treat abscesses, gout, and peptic ulcers as well as an agent for health promotion, stimulation, and vitalization.^12^^,^^13^ In Bangladesh, it has traditionally been utilized as an alternative medicine for diarrhea and vomiting; further, in Laotian folk medicine, it has been valued for its ability to lower glucose levels and boost vitality. Moreover, the rhizome of *K. parviflora*, locally known as Kra-chai-dam in Thailand, has been used to not only treat digestive disorders, oral diseases, and allergies but also—for more than 1000 years—promote longevity, enhance energy expenditure, and improve physical capacity as a form of folk medicine.^14^, ^15^, ^16^ *K. parviflora* reportedly exhibits pharmacological properties, such as anti-osteoporotic,^12^ anti-inflammatory,^13^ anti-oxidant,^17^ and anti-obesity activities.^18^ Additionally, *K. parviflora* extract has been observed to enhance exercise endurance and muscle mass by increasing mitochondrial DNA (mtDNA) content, PGC-1α expression, and the phosphatidylinositol 3-kinase (PI3K)/Akt pathway in animal models.^18^^,^^19^ In particular, a clinical study reported that healthy individuals aged >60 years who took capsuled supplements containing *K. parviflora* extract for 3 months displayed improved physical performance compared with the baseline or placebo-treated group.^17^ However, scientific evidence regarding the anti-sarcopenic activity of *K. parviflora* extract and its specific mechanisms remains lacking. Therefore, this study aimed to explore the inhibitory effects of standardized *K. parviflora* extract (KPE) on sarcopenia in middle-aged mice and clarify the molecular mechanisms underlying protein turnover and mitochondrial homeostasis.

## 2 Materials and methods

### 2.1 Preparation of KPE

Dried *K. parviflora* rhizomes were ground and extracted with 50% ethanol (v/v) at 60°C for 3 h. After filtration, the solvents in KPE were completely evaporated using a rotary evaporator (Laborota 4000; Heidolph Instruments GmbH & Co. KG, Schwabach, Germany), producing a yield of 12.49%. The extract was standardized with its bioactive compound, 5,7-dimethoxyflavone (DMF), using a high-performance liquid chromatography system (YL Instrument Co., Ltd., Anyang, Korea) and Capcell Pak C18 column (250 × 4.6 mm, 5 μm; OSAKA SODA, Osaka, Japan). DMF (>99% purity) was obtained from Indofine Chemical Company, Inc. (Hillsborough, NJ, USA). The analysis was performed under isocratic conditions using MeOH and 0.1% formic acid in water (v/v) at a 66:34 ratio for 30 min. The flow rate and column temperature were set at 0.8 mL/min and 35°C, respectively. The chromatogram was monitored at a wavelength of 254 nm (Supplementary Fig. S1). The DMF content of the standardized KPE was 8.58% (w/w).

### 2.2 Animal experiments

Ten-week-old and eighteen-month-old C57BL/6J mice were supplied by the Laboratory Animal Resource Center (Korea Research Institute of Bioscience and Biotechnology, Cheongju, Korea) and housed in a well-controlled environment (12-h day/night cycles, 25 ± 2°C, and 55 ± 5% humidity) at the Yonsei Laboratory Animal Research Center (Seoul, Korea). All mice had *ad libitum* access to a standard diet and tap water. After 1 week, the older C57BL/6J mice were randomly assigned to one of the following three groups: (I) Aged, (II) 100 mg/kg/day KPE + Aged (KPE100+Aged), and (III) 200 mg/kg/day KPE + Aged (KPE200+Aged). KPE was administered daily via oral gavage for 8 weeks. The Young and Aged groups were provided saline instead of KPE. Body weight was measured once a week. After 8 weeks of treatment, the mice underwent physical performance evaluation and were subsequently anesthetized using 2,2,2-tribromoethanol (Sigma-Aldrich, St. Louis, MO, USA). Under anesthesia, cardiac puncture was performed to collect blood samples. After euthanizing the animals, gastrocnemius, tibialis anterior, soleus, and extensor digitorum longus muscle tissues as well as perirenal, subcutaneous, and visceral fats were carefully excised and weighed. The gastrocnemius muscle was cut into small sections and fixed in 10% formalin (Junsei, Tokyo, Japan). All tissues were stored at −80°C. All experimental procedures were approved by the Institutional Animal Care and Use Committee (IACUC) of Yonsei University (permit number: IACUC-A-201903-874-03).

### 2.3 Treadmill test

An exercise endurance test was performed using a treadmill (LE8710MTS; Panlab, Barcelona, Spain). The test commenced at 10 cm/s for 10 min, followed by 1 cm/s per min increases until the speed had reached 35 cm/s. This rate was maintained until the mice had reached exhaustion, which was defined as the inability to run for 10 s despite a 0.2 mA electric shock.

### 2.4 Grip strength test

Forelimb and fore/hindlimb grip strength tests were conducted using a grip strength meter (Panlab). Each mouse was placed on a grid for visual confirmation of gripping; the mouse's tail was gently pulled back until it released the grid. Five consecutive tests were performed per mouse, and the average value was recorded as the force exerted by the mouse.

### 2.5 Micro-computed tomography (CT) imaging

Micro-CT experiments were conducted using the animal positron emission tomography/CT/single-photon emission tomography system (Siemens Medical Solution, Knoxville, TN, USA). CT images were further analyzed using Inveon Research Workplace software (Siemens Medical Solution).

### 2.6 Histological analysis

Fixed gastrocnemius muscle tissues were paraffin-embedded in blocks, cut into sections, and subsequently mounted on slides. The paraffin slides were stained with hematoxylin and eosin (H&E). Random areas of the stained tissue were captured using the CK40 inverted microscope (Olympus, Tokyo, Japan) equipped with a T500 camera (eXcope, Daejeon, Korea). Mean values of the cross-sectional areas were determined using Image J software (National Institutes of Health, Bethesda, MD, USA). Representative images of the quantified cross-sectional areas are shown.

### 2.7 Reverse transcription-polymerase chain reaction (RT-PCR)

TRIzol™ reagent (Takara, Otsu, Japan) was used to isolate total RNA from gastrocnemius and soleus muscle tissues. The concentration and purity of the RNA were determined using a NanoDrop Lite spectrophotometer (Thermo Fisher Scientific, Inc., Waltham, MA, USA). Complementary DNA (cDNA) synthesis and PCR amplification were performed using the SimpliAmp™ Thermal Cycler (Applied Biosystems, Foster City, CA, USA). RNA was reverse-transcribed to cDNA using RT premix (Elpis Biotech, Daejeon, Korea) at 42°C for 1 h and subsequently at 95°C for 5 min. PCR amplification was performed using the synthesized cDNA, SafeDry Taq PCR premix (CellSafe, Gyeonggi, Korea), and specific genes (Bioneer, Daejeon, Korea). The primer sequences of the genes are shown in Table 1. The thermal cycling protocol was as follows: initial denaturation for 5 min at 95°C, followed by 35 cycles of 30 s at 95°C, 30 s at 58–59°C, and 45 s at 72°C. The final extension step was performed for 5 min at 72°C. The PCR products were stained with Dyne Loading STAR (Dyne Bio, Inc., Daejeon, Korea) and separated on a 1.5% agarose gel. PCR bands were detected using the G:BOX EF imaging system (Syngene, Cambridge, UK) and analyzed with the Gene Snap program. The PCR bands were quantitatively analyzed using Image J software (National Institutes of Health).

**Table 1:** The sequences of forward and reverse primers for target genes.

Gene	Direction	Sequence (5′-3′)
TNF-α	Forward	GAGTCATTGCTCTGTGAAGGGA
	Reverse	ATTCTGAGACAGAGGCAACCTG
IL-6	Forward	AGACAAAGCGAGAGTCCTTCAG
	Reverse	GTCCTTAGCCACTCCTTCTGTG
Catalase	Forward	GGTGCCCCCAACTATTACCC
	Reverse	GAATGTCCGCACCTGAGTGA
SOD	Forward	GACCTGCCTTACGACTATGG
	Reverse	GACCTTGCTCCTTATTGAAGC
GPx	Forward	GTTTGAGAAGTGCGAAGTGAATG
	Reverse	TTAGGTGGAAAGGCATCGGG
PGC-1α	Forward	GAGTGTTCTGGTACCCAAGGC
	Reverse	CTGGGCCGTTTAGTCTTCCTT
NRF-1	Forward	AGATTCGTGGGTGGTAGGGT
	Reverse	TCTAGCAGAGGTCTAGGCGG
Tfam	Forward	AGACTACACTGGGAAACCACAG
	Reverse	GGCTTATAGGGACCCAGTGATG
Beclin-1	Forward	CTCCTTAGGGGATGTTTGCCTT
	Reverse	TCAGGAAAGAGGGAAAGGATGC
LC3	Forward	GGGAGGTCCTGGCTCCTAAA
	Reverse	CAGACAGGCAAGGGCCTAAC
Atg4	Forward	GAGCCTTCCTCCATGTTCTTTCTC
	Reverse	CTCATATCTAGGGGGAGGAAAGGT
Atg7	Forward	TAGAGAGGACCTTGGCCTAACA
	Reverse	CAAGCCATCTATGTGTGTGCTG
MuRF1	Forward	TCTGGACTTAGAACACATAGCAGAG
	Reverse	TCTCCTTCTTCATTGGTGTTCTTCT
Atrogin-1	Forward	CAGTGATCCATTCTGTTCATCCTTG
	Reverse	TTATTTCCAGCCAAATGGAGAGAGA
Mitochondria DNA	Forward	CCGCAAGGGAAAGATGAAAGAC
	Reverse	TCGTTTGGTTTCGGGGTTTC
Genomic DNA	Forward	GCCAGCCTCTCCTGATTTTAGTGT
	Reverse	GGGAACACAAAAGACCTCTTCTGG
β-Actin	Forward	GAAGGAGATTACTGCTCTGGCTC
	Reverse	CTCAGTAACAGTCCGCCTAGAA

### 2.8 Western blot analysis

Gastrocnemius muscle tissues were homogenized with beads in NP-40 Lysis buffer (Elpis Biotech) containing a 0.2% protease inhibitor cocktail (Sigma-Aldrich) for 30 min. The lysates were centrifuged at 16,000×*g* for 15 min and supernatants collected. Supernatant protein concentrations were measured using Bradford solution (Bio-Rad, Hercules, CA, USA), with bovine serum albumin (bioWORLD, Dublin, OH, USA) as the standard reference. Similar amounts of protein in 5 × sample buffer (Elpis Biotech) were boiled for 5 min at 95°C to denature the protein; resolved using 10 or 15% sodium dodecyl sulfate–polyacrylamide gel electrophoresis, depending on the molecular weight of the protein; and transferred to a nitrocellulose membrane (GE Healthcare, Piscataway, NJ, USA). To avoid non-specific binding, the membrane was incubated in 5% skim milk (Difco, Detroit, MI, USA) in Tris-buffered saline (Dyne Bio, Inc.) containing Tween 20. The membrane was exposed to the following primary antibodies against forkhead box O (FoxO)3, phospho (p)-FoxO3, mTOR, p-mTOR, 70-kDa ribosomal protein S6 kinase (p70S6K), p-p70S6K, eukaryotic initiation factor 4E binding protein 1 (4EBP-1), p-4EBP-1, PI3K, p-PI3K, Akt, p-Akt, α-tubulin (Cell Signaling Technology, Beverly, MA, USA), and nuclear factor kappa B (NF-κB) (Santa Cruz Biotechnology, Inc., Santa Cruz, CA, USA) for 18 h at 4°C. Primary antibody incubation was followed by the washing step and subsequent incubation with horseradish peroxidase linked to secondary antibodies (Bethyl Laboratories, Inc., Montgomery, TX, USA) for 2 h at 4°C. Immunoreactive proteins on the membrane were developed using an enhanced chemiluminescence solution (Amersham Biosciences, Little Chalfont, UK) and subsequently detected with the G:BOX EF imaging system (Syngene) using the GeneSys program. Intensities were quantified via densitometry using Image J software (National Institute of Health).

### 2.9 Enzyme-linked immunosorbent assay (ELISA)

Blood samples collected via cardiac puncture under anesthesia were allowed to clot for 5–20 min and centrifuged at 1300×*g* for 15 min, after which serum was collected. ELISA kits (R&D Systems, Minneapolis, MN, USA) were used to measure TNF-α and IL-6 levels, according to the manufacturer's protocols. Absorbance was measured at 540 nm using a microplate reader.

### 2.10 Quantification of mtDNA content

The mtDNA content was measured in the soleus muscle following our previous method.^19^ The primer sequences for mtDNA and genomic DNA (gDNA) are shown in Table 1. The results are expressed as the relative ratio of mtDNA to gDNA determined by the relative density of each band.

### 2.11 Statistical analysis

All data are presented as the mean ± standard deviation. One-way analysis of variance and Duncan's multiple-range test were employed to determine statistically significant differences among groups using SPSS 25.0 software (SPSS Inc., Chicago, IL, USA). Statistical significance of experimental observations was set at *p* < 0.05.

## 3 Results

### 3.1 Effects of KPE on physical performance in aged mice

**Fig. 1:**
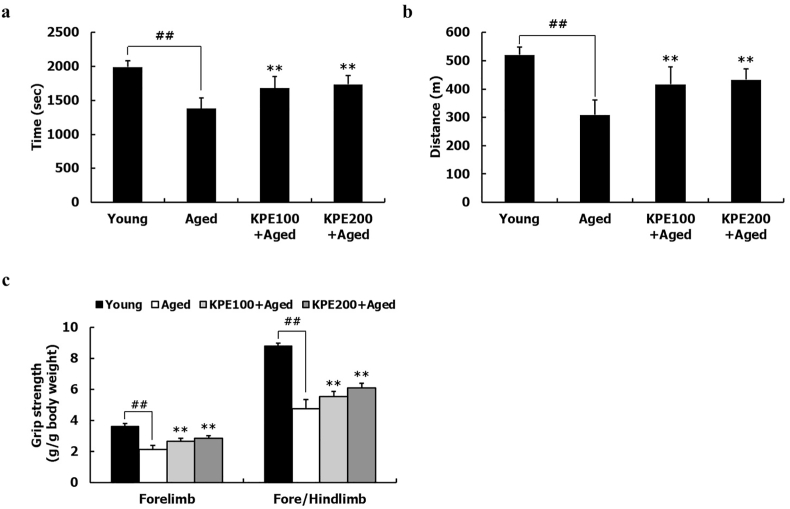
Effects of KPE on grip strength and exercise capacity (a) Running time and (b) running distance were evaluated by treadmill test. (c) Fore/hindlimb and forelimb grip strengths were measured using a grip strength meter. Results are presented as the mean ± standard deviation. ^##^*p* < 0.01 (Young group vs. Aged group); ∗∗*p* < 0.01 (Aged group vs. KPE group).

First, we assessed whether aging and KPE treatment affect physical performance by measuring exercise capacity and grip strength. Decreases in both running time and distance occurred in the Aged group compared with those in the Young group. However, KPE treatment significantly prolonged running time and distance (Fig. 1a and b). The forelimb and fore/hindlimb grip strength values of aged mice were lower than those of young mice. However, KPE treatment significantly raised both strengths in a dose-dependent manner. Oral KPE administration (200 mg/kg/day) increased forelimb and fore/hindlimb grip strengths by 31.84% and 32.85%, respectively (Fig. 1c).

### 3.2 Effects of KPE on body composition in aged mice

**Fig. 2:**
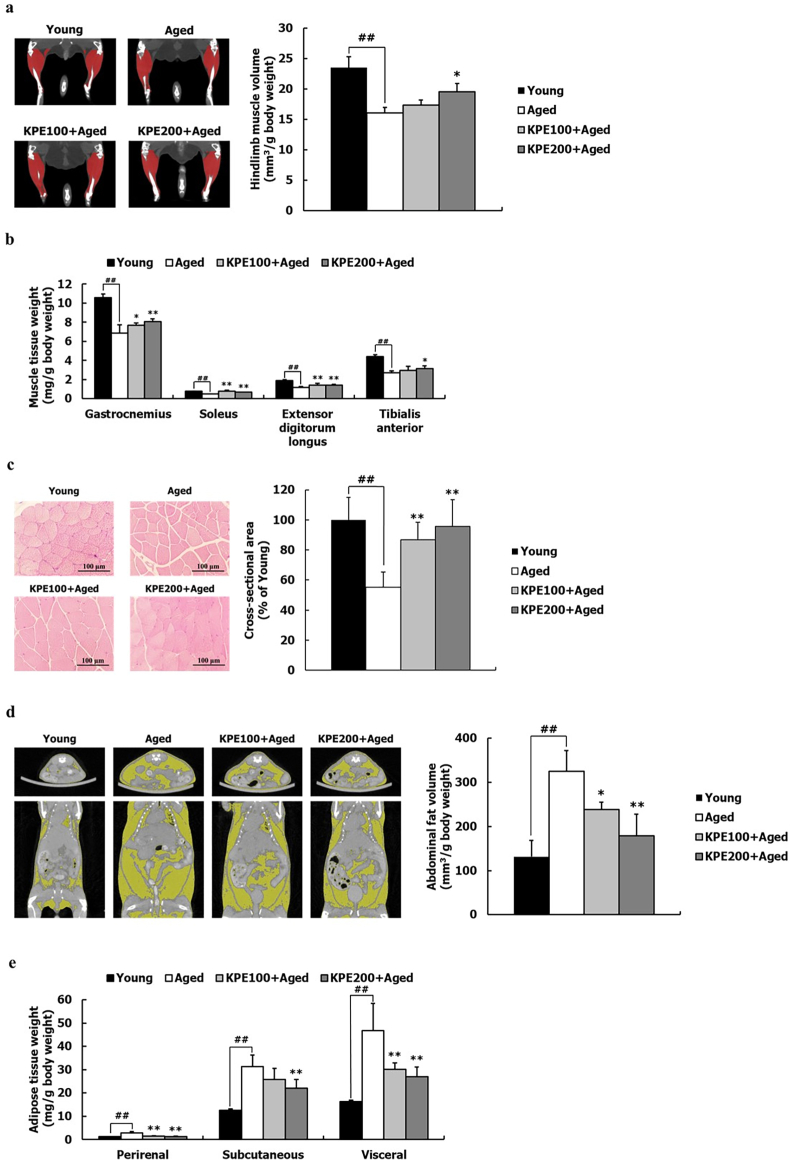
Effects of KPE on muscle and fat volumes and cross-sectional area (a) Hindlimb muscle volume was evaluated using micro-CT. (b) Weights of the gastrocnemius, soleus, extensor digitorum longus, and tibialis anterior muscles were measured. (c) Cross-sectional area of the gastrocnemius muscle fiber was quantified using H&E staining (magnification, × 200). (d) Abdominal fat volume was evaluated using micro-CT. (e) Weights of perirenal, subcutaneous, and visceral adipose tissues were measured. All the tissue weights and volumes were normalized to body weight. Results are presented as the mean ± standard deviation. ^##^*p* < 0.01 (Young group vs. Aged group); ∗*p* < 0.05, ∗∗*p* < 0.01 (Aged group vs. KPE group).

Since body weight significantly differed between young and aged mice (data not shown), tissue weights and volumes were normalized to body weight. We initially measured the total hindlimb-muscle volume for each animal. Compared with that in the Aged group, the ratio of muscle volume to body weight moderately, but not significantly, increased by 8.35% in the KPE100+Aged group and significantly increased by 21.78% in the KPE200+Aged group (Fig. 2a). From the hindlimb, we isolated four muscles: the gastrocnemius, soleus, extensor digitorum longus, and tibialis anterior. Their muscle weight/body weight ratios significantly decreased by 34.96%, 37.48%, 39.10%, and 38.95%, respectively, in the Aged group compared with those in the Young group. KPE treatment dose-dependently increased the gastrocnemius and extensor digitorum longus muscle weight/body weight ratios. In particular, oral KPE administration (200 mg/kg/day) restored 17.33% and 23.15% of the gastrocnemius and extensor digitorum longus muscle weight/body weight ratios, respectively. The soleus muscle weight/body weight ratio exhibited a significant, but not dose-dependent, increase in the KPE+Aged groups. Significant tibialis anterior muscle weight/body weight ratio recovery was exclusively observed in the KPE200+Aged group. Not all muscle weight/body weight ratios were restored to levels similar to those in the Young group (Fig. 2b). Consistent with the muscle volume and weight results, KPE also increased the cross-sectional area of the gastrocnemius muscle in aged mice (Fig. 2c). In this study, we also measured adipose tissue weights, since KPE reportedly exerts anti-obesity effects on obese mice.^18^ The ratio of abdominal fat volume to body weight remarkably increased in the Aged group compared with that in the Young group. However, KPE treatment significantly reduced this ratio by 26.54% and 45.04% in the KPE100+Aged and KPE200+Aged groups, respectively (Fig. 2d). Three adipose tissue weight/body weight ratios in the Aged group markedly exceeded those in the Young group. In the KPE100+Aged group, the perirenal, subcutaneous, and visceral adipose tissue weight/body weight ratios decreased by 48.21%, 17.70%, and 35.53%, respectively. KPE treatment at 200 mg/kg/day significantly reduced the visceral, subcutaneous, and perirenal tissue weight/body weight ratios by 55.91%, 29.67%, and 42.42%, respectively (Fig. 2e).

### 3.3 Effects of KPE on proinflammatory cytokine and anti-oxidant enzyme expression

**Fig. 3:**
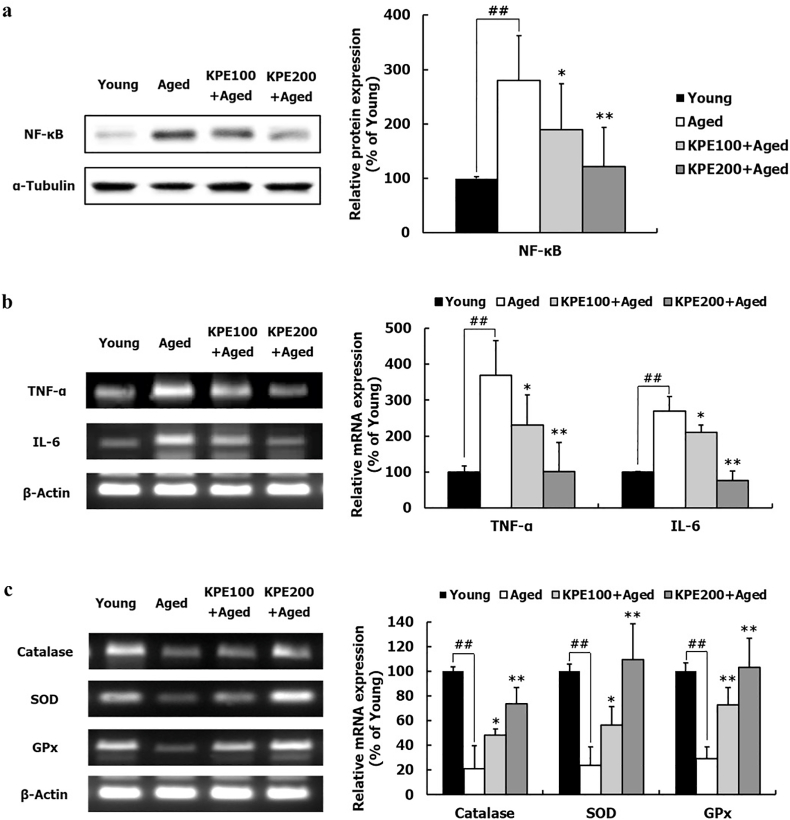
Effects of KPE on inflammatory response in serum and gastrocnemius muscle (a) NF-κB protein expression in the gastrocnemius muscle was analyzed though western blot analysis, with α-tubulin as the housekeeping gene. (b) TNF-α, IL-6, (c) catalase, SOD, and GPx mRNA expression was analyzed through reverse transcription-polymerase chain reaction (RT-PCR), with β-actin as the housekeeping gene. Results are presented as the mean ± standard deviation. ^##^*p* < 0.01 (Young group vs. Aged group); ∗*p* < 0.05, ∗∗*p* < 0.01 (Aged group vs. KPE group).

Serum TNF-α and IL-6 levels are important in patients with sarcopenia because they promote muscle atrophy when they reach muscle fibers via blood circulation.^20^ First, we quantified the serum levels of TNF-α and IL-6 using commercial ELISA kits. Compared with those in the Young group, the serum levels of TNF-α and IL-6 in the Aged group were elevated. However, KPE treatment significantly decreased these elevated levels to values similar to those of the Young group (Table 2). Thereafter, we evaluated changes in the protein expression of NF-κB. The elevated NF-κB protein expression in the Aged group relative to that in the Young group was distinctly reduced by KPE treatment in a concentration-dependent manner (Fig. 3a). Consistently, both TNF-α and IL-6 mRNA expression levels were upregulated in the Aged group but downregulated in the KPE-treated groups (Fig. 3b). In contrast to the patterns of change noted in the inflammatory response-related elements, the mRNA expression levels of anti-oxidant enzymes, catalase, superoxide dismutase (SOD), and glutathione peroxidase (GPx) significantly decreased in the Aged group but increased in the KPE+Aged groups (Fig. 3c).

**Table 2:** Proinflammatory cytokine levels in serum.

Parameter	Young	Aged	KPE100+Aged	KPE200+Aged
TNF-α (pg/mL)	9.94 ± 0.69	33.19 ± 14.12^a^	11.84 ± 2.54^b^	11.17 ± 2.57^b^
IL-6 (pg/mL)	10.05 ± 1.08	40.37 ± 14.79^a^	13.78 ± 3.76^b^	15.73 ± 1.98^b^

KPE100, 100 mg/kg/day *K. parviflora* extract; KPE200, 200 mg/kg/day *K. parviflora* extract.

a *p* < 0.01 vs. Young group.

b *p* < 0.01 vs. Aged group.

### 3.4 Effects of KPE on mitochondrial homeostasis and mitochondrial content

**Fig. 4:**
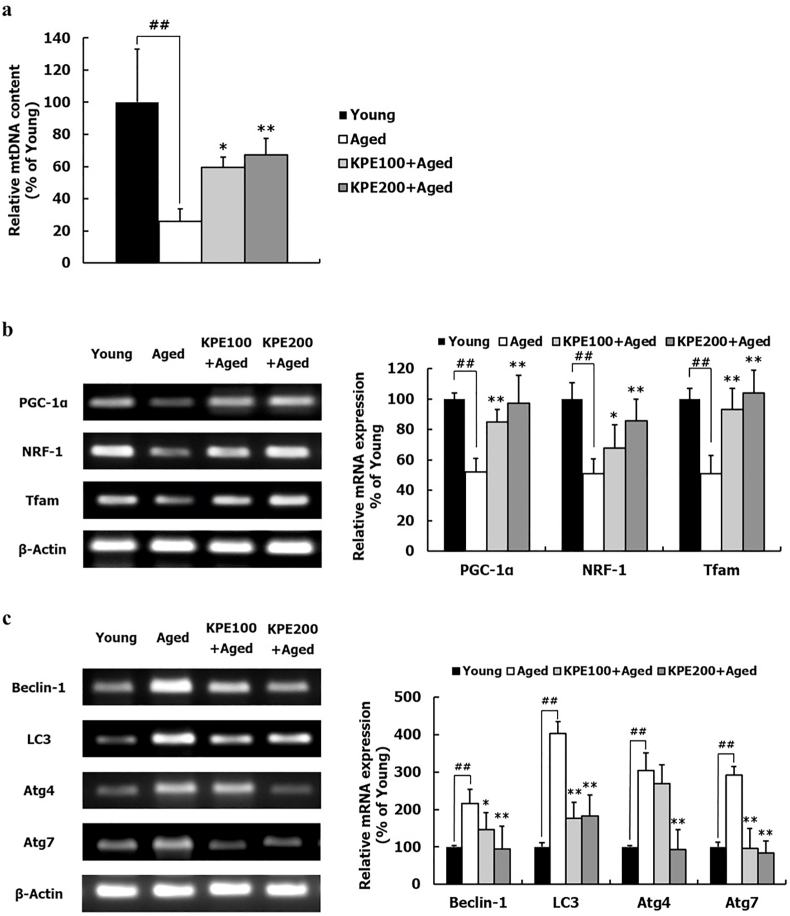
Effects of KPE on mitochondrial contents and mitochondrial homeostasis-related genes in soleus muscle (a) Relative mtDNA content was determined using the mtDNA/gDNA ratio. (b) PGC-1α, NRF1, Tfam, (c) beclin-1, LC3, Atg4, and Atg7 mRNA expression was analyzed through RT-PCR, with β-actin as the housekeeping gene. Results are presented as the mean ± standard deviation. ^##^*p* < 0.01 (Young group vs. Aged group); ∗*p* < 0.05, ∗∗*p* < 0.01 (Aged group vs. KPE group).

We investigated whether KPE increases the mtDNA content of the soleus muscle in aged mice using RT-PCR. Compared with that in the Young group, the soleus muscle in the Aged group possessed less mitochondrial DNA content. KPE treatment increased the mtDNA content in a concentration-dependent manner (Fig. 4a). At the molecular level, the mRNA expression of mitochondrial biogenesis-related genes, PGC-1α, nuclear respiratory factor 1 (NRF-1), and mitochondrial transcription factor A (Tfam) was suppressed in the Aged group; nevertheless, KPE treatment significantly reversed this trend (Fig. 4b). In contrast, the mRNA expression of autophagy-related genes, including Beclin-1, microtubule-associated protein light chain 3 (LC3), autophagy-related protein (Atg)4, and Atg7, were significantly upregulated in the Aged group; however, this upregulation was attenuated by KPE treatment (Fig. 4c).

### 3.5 Effects of KPE on protein turnover-related pathways

**Fig. 5:**
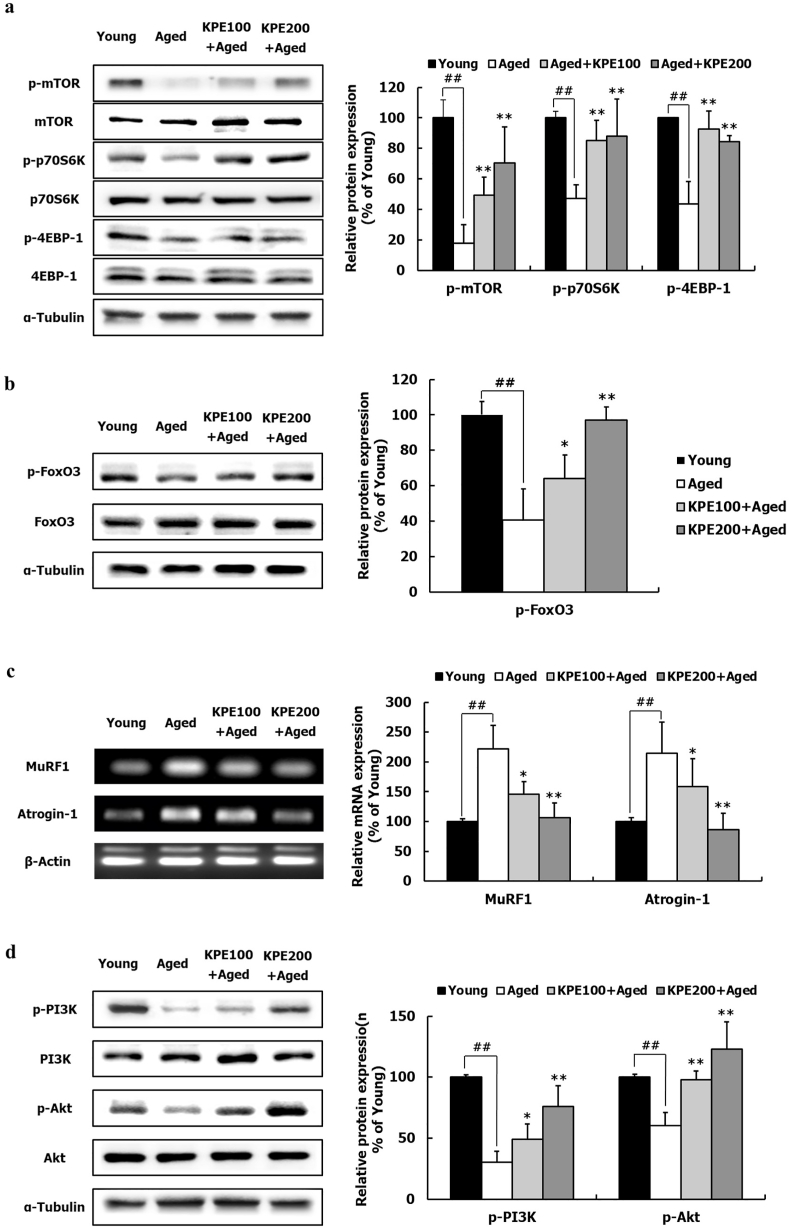
Effects of KPE on protein turnover-related pathway in gastrocnemius muscle (a) p-mTOR, mTOR, p-p70S6K, p70S6K, p-4EBP-1, 4EBP-1, and (b) p-FoxO3a, and FoxO3a protein expression was analyzed though western blot analysis, with α-tubulin as the housekeeping gene. (c) MuRF1 and atrogin-1 mRNA expression was analyzed through reverse transcription-polymerase chain reaction (RT-PCR), with β-actin as the housekeeping gene. (d) p-PI3K, PI3K, p-Akt, and Akt protein expression was analyzed though western blot analysis, with α-tubulin as the housekeeping gene. Results are presented as the mean ± standard deviation. ^#^*p* < 0.05, ^##^*p* < 0.01 (Young group vs. Aged group); ∗*p* < 0.05, ∗∗*p* < 0.01 (Aged group vs. KPE group).

Subsequently, we analyzed protein turnover-related changes in molecular mechanisms in response to KPE treatment and aging in the gastrocnemius muscle. The protein expression levels of p-mTOR, p-p70S6K, and p-4EBP-1 decreased in the Aged group compared with those in the Young group. KPE treatment notably reversed the reduced protein expression of p-mTOR, p-p70S6K, and p-4EBP-1 (Fig. 5a). Once FoxO, which acts as a transcriptional factor, is phosphorylated, it cannot enter the nucleus and regulate target genes, such as muscle ring-finger protein-1 (MuRF1) and muscle atrophy F-box (also called atrogin-1).^21^ The reduced protein expression of p-FoxO3 in the Aged group relative to that in the Young group was dose-dependently upregulated in the KPE+Aged groups (Fig. 5b). Conversely, MuRF1 and atrogin-1 mRNA expression was substantially upregulated in the Aged group compared with that in the Young group; however, KPE treatment evidently reduced this increased mRNA expression (Fig. 5c). These results suggest that KPE prevents FoxO from transcribing MuRF1 and atrogin-1 by stimulating FoxO phosphorylation. We examined the PI3K/Akt pathway, which is a suggested key target for stimulating the protein turnover pathway by activating the mTOR pathway and inactivating FoxO translocation.^21^ The phosphorylated PI3K and Akt content of aged gastrocnemius muscle was lower than that of its young counterpart; nonetheless, KPE treatment significantly restored this content in aged mice (Fig. 5d).

## 4 Discussion

Sarcopenia is a widespread, aging-related muscle disease that has a devastating effect on quality of life and, ultimately, on survival.^1^ The average life expectancy of mice and humans is 24 months and 80 years, respectively; thus 18-month-old mice in the post-senescence phase correspond to humans aged 56–69 years.^22^ To develop interventions targeting the onset and progression of sarcopenia, several studies have used 18-month-old mice, which exhibit inferior muscle mass and physical performance to young mice.^7^^,^^23^ Based on these facts, the current study employed 18-month-old mice. Clinically, sarcopenia is generally diagnosed based on three parameters: muscle mass, physical performance, and strength.^1^ Therefore, grip strength, exercise endurance, muscle mass, and muscle volume were measured as parameters to evaluate the development of sarcopenia in aged mice. The results revealed that these parameters were significantly lower in the Aged group than in the Young group (Fig. 1, Fig. 2a and b), implying that 18-month-old mice present sarcopenic symptoms. *K. parviflora* extract has previously been shown to increase the physical performance of healthy individuals aged >60 years.^17^ In addition, a 95% ethanolic extract of *K. parviflora* was found to attenuate obesity-induced muscle atrophy and increase grip strength and muscle mass in *ob/ob* mice.^18^ Another study demonstrated that *K. parviflora* ethanol extract stimulated exercise endurance and enhanced the skeletal muscle weight/body weight ratio in both normal and high-fat diet-fed mice.^19^ Here, we found 2-month oral administration of 50% ethanol-extracted KPE also increased muscle mass, physical performance, and strength in 18-month-old mice. These results indicate that KPE exerts a protective effect on sarcopenic muscle.

In addition to increased hindlimb muscle mass and volume, the KPE+Aged groups exhibited decreased adipose tissue weight and abdominal fat volume (Fig. 2d and e). Consistently, epididymal and subcutaneous fat weights as well as abdominal fat volume were significantly reduced by *K. parviflora* ethanol extract in obese mice.^18^ Oral 6-week administration of *K. parviflora* dichloromethane extract was shown to decrease epididymal and subcutaneous adipose tissue weights in middle-aged rats.^24^ Notably, no significant differences were observed between the Aged and KPE+Aged groups in terms of body weight (data not shown). Taken together, despite KPE treatment, no net change in body weight was conceivably elicited by the increased muscle mass or reduced adipose tissue weight. Sarcopenic obesity, defined as the presence of increased fat mass and reduced muscle mass in older adults, has recently received increased attention from researchers.^25^^,^^26^ One study proposed an animal model of sarcopenic obesity by providing 18-month-old rats with a high-fat diet; compared with those in normal diet-fed aged rats, the gastrocnemius and tibialis anterior muscles significantly decreased, whereas the perirenal, subcutaneous, and visceral adipose tissues increased in high-fat diet-fed aged rats.^25^ Considering the potential activity of KPE against sarcopenia, the current results indicating that KPE reduces fat weight and volume potentially corroborates the possibility that KPE inhibits the development of sarcopenic obesity in older adults. This hypothesis will be tested by future research focusing on changes in the composition and percentage of fat and muscle tissues relative to body weight, with further clarification of the molecular mechanisms and physiology underlying a sarcopenic obesity animal model.

Systemic and chronic low-grade inflammation is considered a major instigator of sarcopenia.^20^ Proinflammatory cytokines, particularly TNF-α, are potent stimulants of proteolysis via the ubiquitin–proteasome-dependent system.^20^^,^^27^ Therefore, substantial attention has been focused on the anti-inflammatory activities of phytochemicals and nutrients in the development of interventions that mitigate muscle wasting by targeting proinflammatory cytokines.^8^^,^^9^ *K. parviflora* exhibits potent anti-inflammatory activity. TNF-α and IL-6 mRNA expression decreased in the soleus muscle of the *K. parviflora* extract-treated groups after physical performance.^13^ *K. parviflora* also decreased the mRNA expression levels of IL-6 and IL-8 during H_2_O_2_-induced cellular senescence of skin fibroblasts.^28^ Consistently, KPE treatment decreased the plasma and mRNA levels of TNF-α and IL-6 as well as the protein expression of NF-κB in aged muscle (Fig. 3a and b). The anti-oxidant effects of phytochemicals and nutrients are also of interest in the field of sarcopenia,^9^^,^^17^ since TNF-α also gives rise to oxidative stress by producing excess reactive oxygen species, which act as second messengers that not only cause mitochondrial damage but also activate NF-κB, a key mediator of proinflammatory cytokines and MuRF1 transcription.^20^^,^^27^^,^^29^ A previous study indicated that *K. parviflora* extract supplementation increased anti-oxidant enzyme activity and inhibited lipid peroxidation, resulting in enhanced aerobic endurance.^17^ We observed that KPE treatment increased the mRNA expression levels of catalase, SOD, and GPx (Fig. 3c). Along with reducing serum TNF-α and IL-6 levels (Table 2), KPE treatment relieved oxidative stress by increasing anti-oxidant enzyme expression and alleviating inflammatory responses. Therefore, the combined anti-inflammatory and anti-oxidant properties of KPE contribute to its anti-sarcopenic effect.

PGC-1α, a master regulator of mitochondrial biogenesis, is essential for skeletal muscle energy metabolism, fatty acid β-oxidation, and energy substrate transport.^19^^,^^30^ Once PGC-1α interacts with and co-activates NRF-1, mitochondrial biogenesis is stimulated through Tfam, a transcriptional factor responsible for mitochondrial replication and the transcription of genes closely related to mitochondrial function.^30^ Four types of muscle fiber constitute muscle tissue: type I, type IIa, IIb, and IIx. Type I and IIa fibers possess a higher mitochondrial content than the other two fibers, and they compose most of the soleus muscle, which is clearly related to exercise endurance.^31^^,^^32^ In this study, we examined whether KPE enhances mitochondrial biogenesis via the PGC-1α/NRF-1/Tfam pathway using aged soleus muscle. KPE significantly increased the relative mtDNA content and mRNA expression levels of PGC-1α, NRF-1, and Tfam (Fig. 4a and b), indicating that KPE exerts a stimulatory effect on mitochondrial biogenesis through PGC-1α. These findings are supported by previous studies wherein *K. parviflora* increased glycogen levels, mitochondrial content, and the mRNA expression of PGC-1α in obese, normal, and even hairless mice during the intrinsic aging process.^13^^,^^18^^,^^19^^,^^28^ Studies involving C2C12 and L6 myotubes have demonstrated that *K. parviflora* also increased PGC-1α expression, transcriptional activity, and adenosine triphosphate production.^19^^,^^33^ Beclin-1, LC3, Atg4, and Atg7 are key regulators of autophagy, a cellular process requisite to maintaining mitochondrial homeostasis. In the present study, KPE treatment effectively decreased the mRNA expression levels of Beclin-1, LC3, Atg4, and Atg7 (Fig. 4c). These findings suggest that KPE enhances mitochondrial biogenesis and suppresses autophagy, thereby improving sarcopenia.

mTOR is a critical regulator of protein synthesis.^21^ It stimulates protein translation by phosphorylating two downstream factors: p70S6K, which activates the S6 ribosomal protein, and 4EBP-1, which enhances the initiation of mRNA translation.^27^ In contrast to mTOR, FoxO stimulates proteolysis through the ubiquitin–proteasome system by upregulating MuRF1 and atrogin-1.^21^ A previous report revealed an impairment of the mTOR pathway and a substantial increase in the FoxO-regulated ubiquitin–proteasome system in aged muscle.^11^ These findings are supported by the present results wherein the protein expression levels of p-mTOR, p-p70S6K, and p-4EBP-1 were downregulated and those of MuRF1 and atrogin-1 mRNA were considerably upregulated in the aged gastrocnemius muscle compared with those in its young counterpart (Fig. 5a–c). This suggests an imbalance between protein degradation and synthesis in aged mice. Insulin-like growth factor-1 (IGF-1) is a hormone that targets the PI3K/Akt pathway.^21^ The PI3K/Akt pathway, of great interest in developing target agents for sarcopenia, increases protein turnover by stimulating the mTOR pathway and preventing FoxO3 from translocating to the nucleus.^11^ Older people were found to exhibit considerably lower IGF-1 levels, while the PI3K/Akt pathway was significantly impaired in aged mice.^34^ Consistently, reduced p-PI3K and p-Akt protein expression levels were observed in aged mice, indicating that the PI3K/Akt pathway was inactivated. Conversely, KPE not only upregulated p-PI3K and p-Akt protein expression but also downregulated MuRF1 and atrogin-1 mRNA expression, concomitantly increasing the protein expression levels of p-mTOR, p-p70S6K, p-4EBP-1, and p-FoxO3 (Fig. 5). Congruent with these results, another study found KPE activated the PI3K/Akt pathway, inhibiting obesity-induced muscle atrophy.^18^ These results suggest that KPE increases protein turnover by stimulating the PI3K/Akt pathway.

*K. parviflora* contains numerous diverse methoxyflavones. A *K. parviflora*-derived methoxyflavone mixture comprising 5-hydroxy-3,7,3′4′-tetramethoxyflavone, 5-hydroxy-7-methoxyflavone, 5-hydroxy-3,7-dimethoxyflavone, and 5-hydroxy-3,7,4′-trimethoxyflavone was shown to potentially increase the muscle mass and cross-sectional area of the soleus muscle as well as myosin heavy chain type 1 protein expression in SAMP8 mice.^35^ Further, 5-Hydroxy-7-methoxyflavone, 5-hydroxy-3,7,4′-trimethoxyflavone, and DMF reportedly increased PGC-1α mRNA expression in C2C12 myocytes.^33^ In particular, DMF increased muscle mass and physical performance by improving protein turnover and stimulating mitochondrial biogenesis in aged mice.^36^ Not only the methoxyflavones in KPE but also their metabolites (e.g., chrysin, apigenin, and 3-O-methylquercetin)^37^ may augment KPE's inhibitory effect on sarcopenia. For example, apigenin inhibited muscle atrophy in obese mice by improving mitochondrial dysfunction.^10^ Consistent with the current study, *K. parviflora* extract containing 14.4% DMF inhibited muscle atrophy by regulating the PI3K/Akt pathway and mitochondrial biogenesis-related genes in *ob/ob* mice.^18^ Therefore, the anti-sarcopenic effect of KPE is attributable to a mixture of methoxyflavones. However, whether the methoxyflavone mixture in KPE exerts synergistic effects in inhibiting the development of sarcopenia warrants further research.

## 5 Conclusions

In this study, we determined the anti-sarcopenic effect of KPE in aged mice. Oral KPE administration enhanced muscle function, grip strength, and exercise capacity and increased muscle mass, muscle volume, and the cross-sectional area of muscle fibers. Moreover, KPE alleviated inflammatory responses by decreasing proinflammatory cytokines and increasing anti-oxidant enzymes. In terms of mitochondrial quality, KPE stimulated mitochondrial biogenesis, as evidenced by the increased relative mtDNA content and PGC-1α mRNA expression. It also activated the PI3K/Akt pathway, leading to the increased efficiency of protein turnover pathways. These findings provide scientific evidence for the utility of KPE supplementation against sarcopenia. Further studies involving human participants with sarcopenia aged >60 years should be conducted to strengthen these findings and determine the efficacy and safety of KPE in treating sarcopenia.

## Author contributions

C.K: Methodology, Formal analysis, Investigation, Data curation, Writing – Original Draft, Funding acquisition. M.K: Writing – Original Draft, Writing – Review & Editing. Y.K: Writing – Original Draft, Writing – Review & Editing. J.K.H: Conceptualization, Resources, Writing – Original Draft, Writing – Review & Editing, Supervision, Project administration, Funding acquisition.

## Ethical statement

The experimental protocol was approved by the Institute of Animal Care and Use Committee (IACUC) of Yonsei University (Seoul, Korea) (IACUC-A-201903-874-03) to ensure ethical treatment of the animals.

## Data availability

The data that support the findings of this study are included within the article.

## Funding

This work was supported by the Brain Korea 21 (BK21) PLUS program.

## Declaration of competing interest

The authors declare no conflict of interest.
