# Supplementary figures and images for: Inhibitory effect of standardized Kaempferia parviflora extract on sarcopenia by improving protein metabolism pathways in aged C57BL/6J mice
Source: J Tradit Complement Med. 2025 Mar 14;16(4):412–21. doi: 10.1016/j.jtcme.2025.03.007 (PMC13316513; doi:10.1016/j.jtcme.2025.03.007)

**Supplementary Data**

**
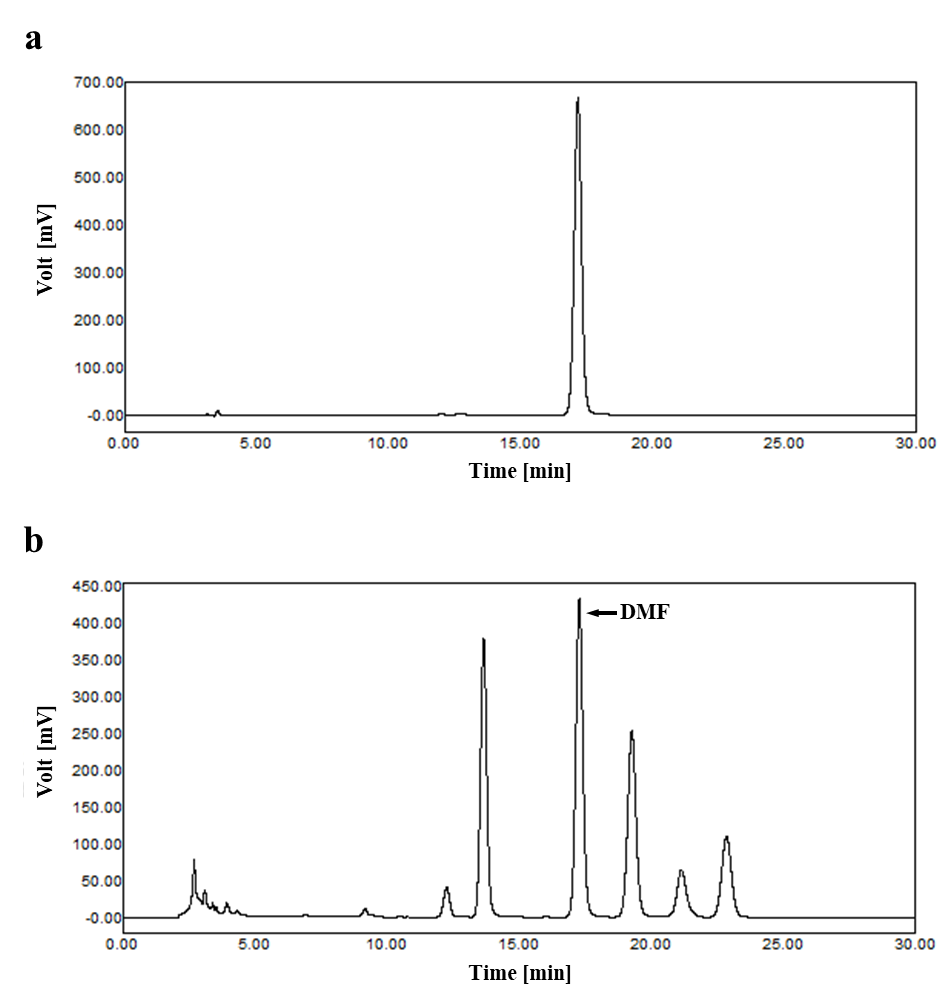
**

Supplementary Fig. S1. The Chromatograms of (a) DMF and (b) KPE.

Supplement: Multimedia component 1 [file mmc1.docx]
